# Long-term smoking cessation rates in elderly versus other adult smokers: A 3-year follow-up study in Taiwan

**DOI:** 10.1016/j.abrep.2018.07.001

**Published:** 2018-07-03

**Authors:** Chiao-Lin Hsu, Kuang-Chieh Hsueh, Ming-Yueh Chou, Hsien-Chung Yu, Guang-Yuan Mar, Hong-Jhe Chen, Robert West

**Affiliations:** a Department of Physical Examination Center, Kaohsiung Veterans General Hospital, Taiwan; b Center for Geriatrics and Gerontology, Kaohsiung Veterans General Hospital, Taiwan; c Department of Family Medicine, Kaohsiung Veterans General Hospital, Taiwan; d Cancer Research UK Health Behaviour Research Centre, Department of Epidemiology and Public Health, University College London; e School of Medicine, National Yang-Ming University, Taiwan; f Division of Gastroenterology, Department of Internal Medicine, Kaohsiung Veterans General Hospital, Kaohsiung, Taiwan; g Division of Cardiology, Department of Internal Medicine, Kaohsiung Veterans General Hospital, Kaohsiung, Taiwan; h Shu-Zen College of Medicine and Management, Kaohsiung, Taiwan

**Keywords:** CPD, cigarettes per day, FTQ, Fagerström Tolerance Questionnaire, FTND, Fagerström Test for Cigarette Dependence, Smoking cessation, Clinic, Transdermal nicotine patch, 3-Year follow-up, Elderly, Older adult

## Abstract

**Introduction:**

Smoking cessation improves life expectancy at any age. There is some evidence that elderly smokers have at least as good a chance of successfully stopping as other smokers but direct comparisons with long-term follow up are rare. This study aimed to compare success rates up to 3 years in smokers aged 65+ versus other adult smokers with and without adjustment for a range of other smoker characteristics.

**Methods:**

This was a prospective study of 1065 smokers who attended a stop-smoking clinic in Taiwan. Participants (896 < 65 years, 169 65+ years) were followed up by telephone 3, 6, 12 and 36 months after the initial quit date. Prolonged abstinence (abstinent at all follow-ups) and point prevalence abstinence (7 days prior to final follow up) were compared between ‘elderly’ participants aged 65+ years versus ‘non-elderly’ participants aged <65 years with and without adjustment for a range of baseline smoker characteristics (sex, educational level, previous quit attempts, cigarette dependence score). Non-responders were considered to be smoking.

**Results:**

Prolonged 36-month abstinence rates were 20.1% (N = 34) and 15.3% (N = 137) in the elderly and non-elderly participants respectively (p = 0.137). Point prevalence 36-month abstinence rates were 37.3% (N = 63) and 26.5% (N = 237) in the elderly and non-elderly participants respectively (p = 0.005). The odds ratios comparing elderly versus non-elderly abstinence rates after adjustment for baseline variables were 1.17 (95%CI = 0.75–1.83) and 1.52 (95%CI = 1.05–2.20) for prolonged abstinence and point prevalence abstinence respectively.

**Conclusions:**

Elderly smokers attending smoker clinics in Taiwan appear to be at least as likely to achieve long-term abstinence as other adult smokers.

## Highlights

• Prolonged 36-month abstinence rates were higher in the elderly group.
• Point prevalence 36-month abstinence rates were also higher in the elderly group. and non-elderly participants respectively.

## 1 Introduction

Smoking cessation has benefits at any age (Burns, 2000; Cataldo, 2003; Doll, Peto, Boreham, & Sutherland, 2004; Ostbye & Taylor, 2004; Taylor, Hasselblad, Henley, Thun, & Sloan, 2002). With aging populations in many countries, the impact of smoking cessation among the elderly is becoming increasingly important (Arai et al., 2012). This raises the question as to whether elderly smokers attending stop-smoking services differ from their younger counterparts in their chances of achieving long-term success at stopping. To address this question, we compared cessation rates 3 years after initial clinic attendance in smokers aged 65+ versus younger smokers.

Age has been found to be a positive predictor of success at stopping in many countries (Vangeli, Stapleton, Smit, Borland, & West, 2011). However, data are lacking on long-term abstinence rates in those attending stop-smoking services. Moreover, it is not clear whether the age gradient in smoking cessation extends to the elderly population. Taiwan has a National Tobacco Smoking Cessation Services Program (Health Promotion Administration, 2016) and is unusual in routinely following attendees up for 3 years. This program provides subsidized smoking cessation medications and behavioral support and thus a rare opportunity to compare long-term smoking cessation rates in elderly and non-elderly smokers receiving smoking cessation treatment.

If elderly smokers are at least as successful as younger smokers at achieving long-term success at quitting, this provides a strong argument for ensuring that they are offered smoking cessation support. There is some evidence that doctors may less likely to advise elderly smokers to stop than younger smokers, perhaps because they assume that they would not succeed or perhaps because they believe that they would not benefit from stopping (Huddlestone, Walker, Hussain-Mills, & Ratschen, 2015). Evidence that elderly smokers are at least as likely to success as younger smokers could mitigate this tendency.

When considering the relative likelihood of success at stopping in elderly and non-elderly smokers, it is important to consider how far this is affected by other factors such as level of nicotine dependence, educational level and past quitting history (Vangeli et al., 2011). It is possible that generational differences in these factors may independently play a role in quit success rates and contribute to any differences observed by age group.

Thus, this study sought to address the question as to whether smokers attending smoking cessation treatment programs differ in long-term abstinence rates from younger counterparts and how far any differences are attributable to other factors.

## 2 Materials and methods

### 2.1 Design and participants

This prospective cohort study was conducted at the Smoking Cessation Clinic, Department of Family Medicine, Kaohsiung Veterans General Hospital, Taiwan, between September 2002 and July 2007. The physicians operating the clinic provided up to eight sessions of counseling and eight weeks of nicotine replacement therapy (nicotine patch). We enrolled participants aged ≥18 years with national health insurance who smoked >10 cigarettes per day (CPD) or had Fagerström Tolerance Questionnaire (Fagerstrom & Schneider, 1989) (FTQ) scores > 5 or Fagerström Test for Cigarette Dependence (Heatherton, Kozlowski, Frecker, & Fagerstrom, 1991) (FTND) scores > 4. They were outpatient who visited family doctors or referred by other multidisciplinary clinicals in our outpatient department or nurses. Others were introduced by hospital employees or volunteers. We excluded smokers who were pregnant and those who had acute or unstable medical conditions. These criteria are set by the Bureau of Health Promotion in Taiwan.

The two difference dependence measures in the study just because of the reimbursement policy in different duration, FTQ was used before 2005 and FTND was used after that time until now.

The cost of therapy for all patients was partially covered by Taiwan's National Insurance Program. The nicotine patch was subsidized NT$250 (approximately US$8.43 or 7.22 €) per week. Participants needed to pay NT$545 (approximately US$18.38 or 15.73 €) per week for the nicotine patch. The study was approved by the Ethics Review Committee of Kaohsiung Veterans General Hospital.

### 2.2 Treatment

We gave all participants 20–30 min smoking cessation counseling, educational materials, and nicotine patches at the first visit. We educated participants how to use the nicotine patch and cope with withdrawal symptoms, explained in detail the “pros” and “cons” of the smoking habit at the first visit. According to the guidelines of the Health Promotion Administration of Taiwan, participants who smoked ≥20 CPD at past three months received Nicotinell TTS30® patches (30 cm^2^; which contains 52.5 mg nicotine and delivers 21 mg nicotine/24 h) for 4 weeks, followed by Nicotinell TTS20® patches (20 cm^2^; which contains 35 mg nicotine and delivers 14 mg nicotine/24 h) for 2 weeks and Nicotinell TTS10® patches (10 cm^2^, which contains 17.5 mg nicotine and delivers 7 mg nicotine/24 h) for 2 weeks. We offered TTS20® patches for 6 weeks and TTS10® patches for 2 weeks to those who smoked <20 CPD. All participants were encouraged, but not required, to revisit the clinic every 1–2 weeks after the first session. We requested them to complete the maximum of eight visits in 90 days. Participants received nicotine patch prescriptions (providing 1–2 weeks' supply) only when they attended the clinic. When participants revisited, we asked them how they were handling withdrawal symptoms and coping with perceived barriers and supplied motivational support.

### 2.3 Study measures

Participants' demographic information including age, sex, education level, daily cigarette consumption, dependence score using Fagerström Tolerance Questionnaire (Fagerstrom & Schneider, 1989) and Fagerström Test for Cigarette Dependence (Heatherton et al., 1991), and previous cessation attempts were assessed at the first visit. The clinic staff recorded all participants' clinic attendance, smoking status, and provision of nicotine patches upon revisit. Participants were contacted by telephone at 3, 6, 12, and 36 months after the first visit to measure their 7-day point abstinence without chemical validation. Participants who did not provide follow-up data or were lost to follow-up were considered to be smokers. The outcome measures were prolonged abstinence (abstinence recorded at all follow up points), and point prevalence abstinence at 36 months.

### 2.4 Statistical analyses

Elderly and non-elderly participants were compared using chi-squared tests for all measures. We also analyzed the number and percent of participants missing from each group for sensitivity analyses. Outcomes were compared between the two groups by logistic regression, adjusting for baseline characteristics: age, sex, educational level, cigarette dependence score and past quit attempts. Daily cigarette consumption was not included because this was a major part of the cigarette dependence score.

## 3 Results

The original study cohort comprised 1096 participants who started treatment during the study period. Thirty-one participants died during the 3-year follow-up period and were excluded from the analysis. Thus, the final cohort comprised 1065 participants. Table 1 shows the characteristics of the two study groups. The elderly group was more likely to be male, had a lower educational level, smoked fewer cigarettes per day, had lower cigarette dependence scores, attended more sessions and used nicotine replacement therapy for longer. There were non-significantly more likely to demonstrate prolonged abstinence and significantly more likely to show 7-day point prevalence abstinence at 36 months. On the other hand, the number and percentage of participants missing are 55 (32.5%) for elderly group and 306 (34.2%) for non-elderly group, p value = 0.686, there were non-significantly. In addition, we reanalysis the complete data treating missing as missing for 36 months 7-days abstinence rate and reveal abstinence rate are 55.3% (63) for elderly group and 40.2% (237) for non-elderly group, p value = 0.003, the 7-day point prevalence abstinence at 36 months still higher in elderly group than non-elderly group (Table 1).

**Table 1:** Characteristics of elderly and non-elderly participants.

	Elderly	Non-elderly	Total	p-Value for difference
Percent (N) male	93.5 (158)	87.3 (782)	88.3 (940)	0.019^a^, ⁎
Mean (SD) age	73.8 (5.07)	40.2 (10.95)	45.6 (15.98)	<0.001^b^, ⁎
Educational level				<0.001^c^, ⁎
percent (N)				
Primary school	22.5 (38)	3.9 (35)	6.9 (73)	
High school	42.6 (72)	46.0 (412)	45.4 (484)	
College/university	26.6 (45)	45.9 (411)	42.8 (456)	
Unknown	8.3 (14)	4.2 (38)	4.9 (52)	
Mean (SD) daily cigarette consumption	21.6 (11.68)	25.2 (12.16)	24.6 (12.15)	<0.001^b^, ⁎
Mean (SD) FTCD	5.8 (2.10)	6.5 (2.22)	6.4 (2.21)	<0.001^b^, ⁎
Percent (N) tried to quit previously	63.9 (108)	63.1 (565)	63.2 (673)	0.862^a^
Mean (SD) sessions attended	3.6 (2.49)	2.9 (2.17)	3.0 (2.24)	0.001^b^, ⁎
Mean (SD) weeks of nicotine patch use	4.0 (2.72)	3.4 (2.47)	3.5 (2.52)	0.002^b^⁎
Percent (N) 7-day point prevalence abstinence at 36 months	37.3 (63)	26.5 (237)	28.2 (300)	0.005^a^, ⁎
Percent (N) lost follow up participants^d^	32.5(55)	34.2 (306)	33.9(361)	0.686
Percent (N) 7-day point prevalence abstinence at 36 m (without lost fu)	55.3 (63)	40.2 (237)	42.6 (300)	0.003^c^, ⁎
Percent (N) prolonged abstinence at 36 months	20.1 (34)	15.3 (137)	16.1 (171)	0.137^a^

a Fisher's exact test.

b Analysis of variance.

c Chi-squared test.

d Lost follow up participants are calculated as smokers in logistic regression.

⁎ p < 0.05 represented significant differences between elderly and non-elderly group.

Table 2 shows the result from the logistic regression analyses. Adjusting for baseline characteristics, elderly smokers were slightly and non-significantly likely to show higher prolonged abstinence and significantly more likely to show higher point-prevalence abstinence at 36 months. Women were less likely to be abstinent (by both outcome measures) than men, and abstinence was negatively associated with nicotine dependence score. Past quit attempts, being male and lower cigarette dependence score were all positively associated with both measures of abstinence.

**Table 2:** Results of logistic regression analyses predicting abstinence.

	Prolonged abstinence at 36 months	Point-prevalence abstinence at 36 months
Sex (ref = male)	0.41 (0.21–0.82)^a^	0.46 (0.28–0.77)^a^
Educational level		
Primary school (ref)	–	–
High school	0.69 (0.36–1.33)	1.08 (0.60–1.95)
College/university	0.67 (0.34–1.30)	1.10 (0.61–2.00)
Unknown	0.37 (0.13–1.08)	1.00 (0.44–2.26)
Cigarette dependence	0.89 (0.83–0.96)^a^	0.90 (0.84–0.95)^a^
Tried to quit previously (ref = no experience)	1.63 (1.13–2.36)^a^	1.55 (1.15–2.08)^a^
Age group (ref = non-elderly)	1.17 (0.75–1.83)	1.52 (1.05–2.20)^a^

a Represented significant differences between elderly and non-elderly group.

The reason of smoking cessation between two groups is shown in Table 3. The reasons for smoking cessation have excluded 26 missing data, including 22(2.5%) for non-elderly and 4 (2.4%) for elderly. There is no statistically significance (p = 0.945). The most common reason for smoking cessation among two groups was for health or afraid of being illness in the future. Which include 128(77.6) in elderly participants and 774(88.6) in non-elderly participants. The second reason was for their family, 64(38.8) in elderly group and 502(57.4) in non-elderly group. However, the third reason for elderly participants was according to doctors' suggestion. Economic concern was the third smoking cessation reason for non-elderly participants.

**Table 3:** Reasons for smoking cessation.

	Elderly	Non-elderly	Total	p-Value for difference
Total participants	165	874	1039	
Reasons for quit^a^				
For health	128 (77.6)	774 (88.6)		<0.001^b^
For family	64 (38.8)	502 (57.4)		<0.001^b^
For job	0 (0)	113 (12.9)		<0.001^b^
Doctor suggestion	41 (24.8)	121 (13.8)		<0.001^b^
For money	13 (7.9)	172 (19.7)		<0.001^b^
For friend	17 (10.3)	170 (19.5)		<0.001^b^

a Reasons for quit have excluded 26 missing data: 22 (2.5%) for non-elderly and 4 (2.4%) for elderly, no statistic significant (p = 0.945).

b Chi-squared test.

## 4 Discussion

Taiwanese smokers aged 65+ were slightly and non-significantly more likely to show prolonged abstinence, and significantly more likely to show point-prevalence abstinence 36 months after attending a smoking cessation treatment program than younger smokers. This pattern of results was not affected by baseline characteristics such as nicotine dependence.

The results suggest that elderly smokers potentially benefit at least as much from smoking cessation treatment as other smokers in terms of abstinence. The cessation rates are relatively high compared with what is observed in clinical trials (Cawkwell, Blaum, & Sherman, 2015). This is despite relatively high cigarette consumption and cigarette dependence scores.

The fact that the difference between elderly and non-elderly smokers was significant for point-prevalence but not for prolonged abstinence suggests that elderly smokers are more likely to succeed in quitting over time than their younger counterparts, either because they make more quit attempts, or because those quit attempts are more likely to succeed. Clearly smoking cessation remains a priority for elderly people even if they relapse following a course of treatment.

The higher abstinence rates in men than women coincides with some findings (Perkins & Scott, 2008; Vogel et al., 2014) in the literature but not others (Munafo, Bradburn, Bowes, & David, 2004; Wetter et al., 1999). Smoking is rare in Taiwanese women and it is possible that those who become smokers have a greater propensity to become dependent, whereas in western societies with more equal smoking prevalence between the sexes, this factor is less evident.

The elderly smokers appeared to engage to a greater extent with the treatment programme, attending more sessions and using nicotine replacement therapy for more weeks. This suggests that elderly smokers who attend smoking treatment programs are motivated to succeed and benefit from the treatment on offer.

We found the highest proportion of smokers with cessation reasons reported the “health problems” as their primary motivators for quitting smoking in two groups. This is the same with another Japan cross-sectional study (Tanihara & Momose, 2015). One previous study has shown that older smokers are more likely to experience health problems and often reported as a reason for stopping smoking (Osler & Prescott, 1998). Instead of economic consideration, we found that the third common reason for smoking cessation in elderly participants is for their family. Despite some literature found increased cigarette prices was associated with higher quitting rate (Mayne et al., 2017; Mayne et al., 2018), even in elderly (Stevens et al., 2017). However, we observed this condition is more apparent in non-elderly group. These findings implying that subjects could be motivated to stop smoking by individualized consideration.

The study was limited by the fact that abstinence was not verified biochemically. This may have inflated the overall success rate, but it is not clear that falsely claiming abstinence would be more likely among elderly participants. A second limitation was that data were drawn from one clinic within Taiwan and it is possible that the results were specific to this clinic. However, the clinic was run according to the standards set nationally and it is difficult to think of reasons why it should be special in terms of the characteristics of elderly versus non-elderly smokers attending.

Overall, this study found that long-term abstinence rates were at least as high in elderly Taiwanese smokers attending smoking cessation treatment than in non-elderly smokers. This provides reassurance that such services meet the needs of this important and growing population.
